# PaWFE: Fast Signal Feature Extraction Using Parallel Time Windows

**DOI:** 10.3389/fnbot.2019.00074

**Published:** 2019-09-10

**Authors:** Manfredo Atzori, Henning Müller

**Affiliations:** ^1^Information Systems Institute, University of Applied Sciences Western Switzerland (HES-SO Valais), Sierre, Switzerland; ^2^University of Geneva, Geneva, Switzerland

**Keywords:** surface electromyography, hand prosthetics, feature extraction, classification, signal processing, machine learning

## Abstract

**Motivation:** Hand amputations can dramatically affect the quality of life of a person. Researchers are developing surface electromyography and machine learning solutions to control dexterous and robotic prosthetic hands, however long computational times can slow down this process.

**Objective:** This paper aims at creating a fast signal feature extraction algorithm that can extract widely used features and allow researchers to easily add new ones.

**Methods:** PaWFE (Parallel Window Feature Extractor) extracts the signal features from several time windows in parallel. The MATLAB code is publicly available and supports several time domain and frequency features. The code was tested and benchmarked using 1,2,4,8,16,32, and 48 threads on a server with four Xeon E7- 4820 and 128 GB RAM using the first 5 datasets of the Ninapro database, that are recorded with different acquisition setups.

**Results:** The parallel time window analysis approach allows to reduce the computational time up to 20 times when using 32 cores, showing a very good scalability. Signal features can be extracted in few seconds from an entire data acquisition and in <100 ms from a single time window, easily reducing of up to over 15 times the feature extraction procedure in comparison to traditional approaches. The code allows users to easily add new signal feature extraction scripts, that can be added to the code and on the Ninapro website upon request.

**Significance:** The code allows researchers in machine learning and biosignals data analysis to easily and quickly test modern machine learning approaches on big datasets and it can be used as a resource for real time data analysis too.

## Introduction

Hand amputations can dramatically affect the quality of life of a person. The combination of surface electromyography and machine learning is a promising solution to control dexterous robotic hands. However low control robustness, intuitiveness and adaptivity prevent the advent of prosthetic hands that can be controlled naturally, like real hands, in real life settings (Micera et al., 2010; Farina et al., 2014; Atzori and Müller, 2015).

Worldwide research groups are working to make machine learning algorithms capable to analyze electromyography data for hand prosthetics in real time and robustly. Real time control experiments provide the best evaluation of prosthesis usability (Hargrove et al., 2007; Scheme and Englehart, 2011). However, these studies require the interaction of the user with the control system, so they do not allow to easily compare new analysis procedures (unless the entire study is repeated) (Pizzolato et al., 2017). Offline experiments allow to easily test and compare new methods but can take several weeks of computational time.

Many control approaches have been tested and applied, both in commercial applications and in scientific research (Farina et al., 2014; Atzori et al., 2016a). Among those, the classification approach described by Englehart and Hudgins (2003) stands out for simplicity and wide use. This approach is based on continuous, windowing-based signal classification and it can lead to high accuracy (Peerdeman et al., 2011).

Publicly available benchmark datasets and software have been released, in order to foster data analysis and to compare various methods and setups. The biggest publicly available benchmark database is NinaPro, which currently includes hand movement sEMG data acquisitions from over 130 intact and amputated subjects (Atzori et al., 2014b; Krasoulis et al., 2017; Palermo et al., 2017; Pizzolato et al., 2017).

Offline feature extraction of big sEMG datasets can easily take several weeks of computational time.

Several studies targeted feature extraction in sEMG. However, to our knowledge there is no software available to extract signal features in parallel windows. Examples of publicly available code to run sEMG data analyses include: Biopatrec, a MATLAB-based research platform for the control of artificial limbs based on pattern recognition algorithms (Ortiz-Catalan et al., 2013); the Myoelectric Control Development Toolbox^1^, a set of MATLAB scripts for myoelectric control (Chan and Green, 2017); The BioSig project, an open source library for bioelectric signal processing^2^; Bio-SP tool (Nabian et al., 2018); Physiolab (Muñoz et al., 2018). Currently available code is useful for many different applications, including real time data analysis (Ortiz-Catalan et al., 2013). However, the mentioned algorithms do not extract signal features in parallel time windows. Thus, feature extraction from big datasets (such as Ninapro) can easily take several weeks of computational time.

PaWFE (Parallel Window Feature Extractor) solves this problem by consistently reducing the computational time required to perform feature extraction, allowing researchers to perform scientific research faster. The code can easily reduce over 15 times the feature extraction computational time, which is related to the hardware. The approach is based on window thread parallelization. The code is developed in MATLAB, it easily allows to extract in few seconds common signal features (including the ones described in Chan and Green, 2017) and it easily allows users to include new features into the workflow.

In this paper, the performance of the code is also compared with two widely used feature extraction algorithms: BioPatRec, (Ortiz-Catalan et al., 2013) and the Myoelectric Control Development Toolbox^3^ (Chan and Green, 2017), showing that the proposed approach is effective in reducing the feature extraction time.

The signal feature extraction scripts were used in previous works on sEMG data analysis and on kinematics data too. In particular, their application to both sEMG and kinematic data allowed to create recently a quantitative taxonomy of hand movements based on both muscular and kinematic information (Stival et al., 2019).

The parallel signal feature extraction scripts were tested on sEMG data in this paper. However, they can also be useful for the analysis of other biosignals, such as electroencephalogram (EEG), electrocorticogram (ECoG), electrocardiogram (ECG), electrooculogram (EOG) or kinematics.

## Methods

### Parallel Feature Extraction Algorithm

The parallel signal feature extraction code presented in this paper aims at reproducing the feature extraction part of the signal classification procedure described by Englehart and Hudgins (2003) using parallel multiple cores, in order to reduce computational time. This signal classification procedure is often used in studies targeting real time classification of surface electromyography signals, as well as offline data analysis. The approach consists of windowing, signal feature extraction and signal feature classification. Offline analyses usually use part of the recorded movement repetitions for training and the remaining ones for testing. The method has several advantages in comparison to other approaches: it does not require segmentation of the sEMG data; it allows delivering a continuous stream of class decisions to the prosthesis; it allows substantial gains in classification accuracy and response time; it allows natural control without interruption and it requires minimal storage capacity for real time approaches, which is an important factor in embedded control systems. Due to the mentioned advantages, this approach is often used in recent online and offline studies targeting the classification of surface electromyography data for natural control of robotic hand prostheses.

The code presented in this paper is developed in MATLAB (MathWorks Inc., Natick, MA) and is publicly available on the Ninapro website^4^. The code allows to accelerate the signal classification procedure described by Englehart in machines with multiple cores by analyzing time windows in separate different threads. Therefore, the approach can be useful to accelerate both offline and online data analyses, also allowing to use better performing but more complex signal features. In its final version, the code has dependencies on functions from specific MATLAB toolboxes as well as from the pattern recognition library developed by Chan and Green (2017), which provides the scripts for the extraction of some features (adapted in some cases to the code requirements).

**Figure 1:**
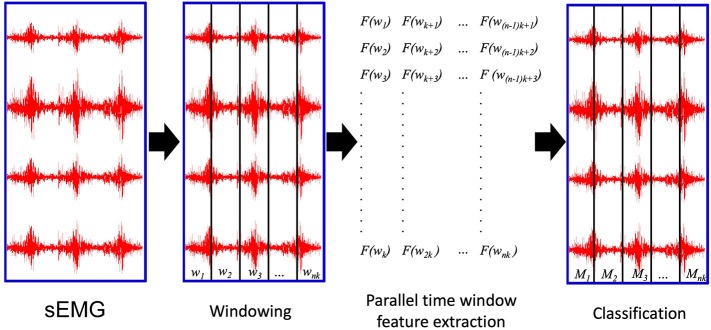
The parallel time window feature extraction: w_i_ are the time windows to be analyzed; *k* is the number of parallel threads used to compute the signal features. *nk* is the total number of time windows; M_i_ are the movements classified according to the extracted signal features.

The PaWFE workflow is represented in Figure 1. As represented in the flow chart, the sEMG signal is first divided in time windows. Afterwards, the function to compute the signal feature is run in parallel on a number of time windows that corresponds to the number of threads that the user decided to open (*k* in the figure). The process is completed once the signal is fully analyzed. The main code function requires the following input variables: *emg, stimulus, repetition, deadzone, winsize, wininc, featFunc, ker*. The input variable *emg* is the electromyographic signal. It is expected to be an *m x n* matrix where each column represents the signal provided by an electrode while each row represents the synchronized time samples of all the electrodes. The input variable *stimulus* represents the movement repeated by the subject. It is expected to be a column vector of integers, each corresponding to a specific movement that can be repeated several times. This value is fundamental to allow the classification of the movements. Using Ninapro, both labeled or relabeled data can be used. The input variable *repetition* represents the repetition of the movement, which can be important in offline studies to select the movements for the training and testing set. The input variable *deadzone* is the positive and negative limit that the signal or the slope must cross to be considered a dead zone in the zero crossing and the slope sign change features. This value is also required for the set of time domain statistics feature. The input variable *winsize* is the length of the time window to be analyzed. It is expressed in terms of samples, so it is equal to the sampling frequency multiplied by the length of the time window in seconds. The input variable *wininc* is the increment of the sliding window. Also in this case, the value is equal to the sampling frequency multiplied by the increment expressed in seconds. The input variable *featFunc* is the feature to be extracted. Currently, PaWFE allows to extract (for each signal x and time window w of T samples) 10 features, including: Integrated Absolute Value, Mean Absolute Value, Slope Sign Change, Zero Crossing, Mean Absolute Value Slope, Root Mean Square, Waveform Length, Histogram, marginal Discrete Wavelet Transform and a complete set of time domain statistics widely used in literature.

Integrated Absolute Value, IAV (input string: getiavfeat) (Zardoshti-Kermani et al., 1995):

$$\begin{array}{l} IAV_{w}\left(x\right)=\overset{T}{\underset{t=1}{\sum }}|x_{t}| \end{array}$$

Mean Absolute Value, MAV (getmavfeat) (Hudgins et al., 1993):

$$\begin{array}{l} MAV_{w}\left(x\right)=\frac{1}{T}\overset{T}{\underset{t=1}{\sum }}|x_{t}| \end{array}$$

Slope Sign Change SSC (getsscfeat) (Hudgins et al., 1993), defined as the number of times the sign of the slope changes. The SSC of a signal *x* in a given window *w, SSCw*(*x*), is incremented if, given three consecutive samples *x*_*t*__−1_, *x*_*t*_ and *x*_*t*__+1_, {*x*_*t*_ > *x*_*t*__−1_ and *x*_*t*_>*x*_*t*__+1_} or {*x*_*t*_<*x*_*t*__−1_ and *x*_*t*_<*x*_*t*__+1_} and {|*x*_*t*_ – *x*_*t*__+1_| ≥ threshold or |*x*_*t*_ – *x*_*t*__−1_| ≥ threshold}.

Zero Crossing, ZC (getzcfeat) (Hudgins et al., 1993), obtained by increasing the feature value by one if, given two consecutive samples *x*_*t*_ and *x*_*t*__+1_, {*x*_*t*_ > 0 and *x*_*t*__+1_ <0} or {*x*_*t*_ <0 and *x*_*t*__+1_ > 0} and |*x*_*t*_ – *x*_*t*__+1_| ≥ threshold.

Mean Absolute Value Slope, MAVS (getmavsfeat) (Hudgins et al., 1993):

$$\begin{array}{l} MAVS_{w}\left(x\right)=MAV_{w+1}\left(x\right)-MAV_{w}\left(x\right) \end{array}$$

Root Mean Square, RMS (getrmsfeat) (De Luca, 1997), where *x*_*t*_ is the *t*^*th*^ sample in the window *w*:

$$\begin{array}{l} RMS_{w}\left(x\right)=\sqrt{\frac{1}{T}\sum_{t=1}^{T}x_{t}^{2}} \end{array}$$

Waveform length, WL (getwlfeat) (Hudgins et al., 1993):

$$\begin{array}{l} WL_{w}\left(x\right)=\sum_{t=2}^{T}|x_{t}-x_{t-1}| \end{array}$$

The histogram (HIST) signal feature (Zardoshti-Kermani et al., 1995) obtained by dividing a 3σ threshold into B = 20 bins (getHISTfeat):

$$\begin{array}{l} HIST\left(x\right)=hist\left(x_{1:T},B\right) \end{array}$$

The marginal Discrete Wavelet Transform (Lucas et al., 2008) created with a db7 wavelet with 3 levels (getmDWTfeat). In the formula, ψ_l, τ_ denotes the mother wavelet with translation l dilation τ.

$$\begin{array}{l} mDWT_{l}\left(x\right)=\sum_{\tau =0}^{T/2^{l}-1}\left|\sum_{t=1}^{T}x_{t}\psi_{l,\tau }\left(t\right)\right|\\ \;\psi_{l,\tau }\left(t\right)=2^{-\frac{m}{2}}\psi \left(2^{-l}t-\tau \right) \end{array}$$

The set of time domain statistics described in detail in the paper by Hudgins et al. (1993), TD (which includes the concatenation of MAV, MAVS, ZC, SSC and WL, getTDfeat).

Finally, the input variable *ker* corresponds to the number of CPU cores to be used for the computation.

The code outputs the following variables: *feat, featStim*, and *featRep*. The output variable *feat* corresponds to the features extracted. It has a number of rows equal to the number of extracted time windows and a number of columns which depends on the dimension of each signal feature. The output variable *featStim* provides the input variable *stim* that corresponds to each time window. The output variable *featRep* provides the input variable *repetition* that corresponds to each time window. Time windows that do not have a unique value for *stim* or *repetition* variables are removed from the output variables.

### Algorithm Validation Experiments

The experiments validate the parallel feature extraction algorithms on 5 datasets recorded with varying acquisition setups. In particular, they measure how much the parallel window feature extraction procedure reduces the feature extraction time using an increasing the number of threads and they verify that the extracted features can lead to classification performance that are comparable to the results described in scientific literature.

#### Datasets

The data include 50 subjects from the Ninapro database, a publicly available database of electromyography, kinematics and dynamic data related to hand movements. Currently, Ninapro includes data acquisitions from 117 intact subjects and 13 trans radial amputees including multimodal signals. In order to take the variability of the scripts in relation to different subjects and acquisition setups into account, we considered the first 10 subjects from each of the first 5 Ninapro datasets, therefore including 40 intact subjects and 10 transradial amputees. All participants signed an informed consent form and the experiments of the data acquisition were approved by the Ethics Commission of the state of Valais (Switzerland). The datasets are publicly available in the Ninapro database^5^ and thoroughly described in the corresponding reference papers (Atzori et al., 2014a, 2016b; Krasoulis et al., 2017; Palermo et al., 2017; Pizzolato et al., 2017). Each dataset contains files for each subject and exercise in MATLAB format with filtered and synchronized data.

#### Acquisition Protocol

The subjects imitated several repetitions of hand movements that were shown on the screen of a laptop as movies. Intact subjects were asked to imitate the movements with the right hand, while amputated subjects were asked to think to imitate the movements with the missing hand, as naturally as possible. In order to obtain comparable results, the same hand movements are considered in all the datasets (Ninapro exercise B and C plus rest, 41 hand movements in total). The acquisition protocol included 10 movement repetition for dataset 1, 6 repetitions for dataset 2, 3, 4, and 5. Movement repetitions lasted 5 s and were followed by 3 s of rest.

#### Acquisition Setups

The 5 datasets were recorded with 4 acquisition setups that allowed to record several multimodal signals, such as surface electromyography, acceleration, kinematics and force. The description of the sEMG acquisition setups is summarized here for completeness, while more thorough descriptions can be found in the datasets reference papers (Atzori et al., 2014a, 2016b; Krasoulis et al., 2017; Palermo et al., 2017; Pizzolato et al., 2017). In the Ninapro DB1, the muscular activity of the subjects was recorded with ten double differential electrodes (OttoBock MyoBock 13E200-50, Otto Bock HealthCare GmbH^6^), providing an amplified, bandpass-filtered and root mean square rectified version of the raw sEMG signal at 100 Hz. An elastic armband was used to keep the electrodes attached to the skin of the subjects and the amplification level was set to 5. Eight sensors were placed equally spaced around the forearm at the height of the radio-humeral joint and two sensors were placed on the main activity spots of the flexor and extensor digitorum superficialis (identified by palpation) (Atzori et al., 2014a,b). In the Ninapro DB2 and DB3 (Atzori et al., 2014a, 2016b), the electromyographic activity of the subjects was recorded with 12 Delsys Trigno Wireless System^7^ double differential electrodes, that provide raw sEMG signal at 2 kHz. The Trigno standard adhesive bands and an hypoallergenic elastic latex–free band were used to keep the electrodes attached to the skin of the subjects. Eight sensors were placed around the forearm at the height of the radio-humeral joint and two sensors were placed on the main activity spots of the flexor and extensor digitorum superficialis. Two more sensors were placed on the main activity spots of the biceps brachii and triceps brachii. The main activity spots were identified by palpation. In Ninapro DB4, the muscular activity of the subjects was recorded with a Cometa Wave Plus double differential wireless system using the miniWave sensors^8^, providing 2 kHz signal at 16 bit sampling rate. The electrodes were placed following the protocol already used for the Ninapro DB2 and DB3 datasets. In this case the subjects were shaved, scraped and disinfected on the electrode spots. In the Ninapro DB5, the electromyographic activity of the subjects was recorded with two Thalmic Myo armbands^9^, each including 8 sEMG single differential electrodes providing 200 Hz signals with a resolution of 8 bit unsigned via Bluetooth. The two Myo armbands are worn one next to the other. The upper one is placed closer to the elbow with the first electrode on the radio humeral joint, while the lower one is set just below the first, tilted in order to fill the gaps left by the other Myo. This configuration provides a uniform muscle mapping with performance comparable to very expensive acquisition setups at extremely affordable prices. The data recorded from the two armbands can be analyzed together or separately (Pizzolato et al., 2017). Power line interference can affect feature extraction. The Otto Bock electrodes and the Thalmic Myo armband adopt strategies to avoid problems related to it (frequency shielding and filtering). The Delsys Trigno and the Cometa sensors on the other hand are not shielded against interference. Therefore, their signal was filtered offline using a Hampel filter (Atzori et al., 2014a). The movements performed by the subjects can begin and end at different timings from the original stimuli, therefore offline relabeling was performed (Atzori et al., 2014a; Pizzolato et al., 2017).

#### Feature Extraction

Signal features are extracted both with PaWFE and with BioPatRec, in order to compare the outcoming computation times and classification accuracy. When using PaWFE, the first three input variables provided are constant among the datasets: emg, relabeled movement stimulus, relabeled movement repetition. The *deadzone* input variable was set to the following values, determined after several experimental tests: 10^−5^ for DB1, DB2, and DB3; 10^−3^ for DB4; 10 for DB5. The variables *winsize, wininc* were respectively set to the equivalent of 200 ms and 10 ms in terms of time samples. Such values often used in scientific literature since they can correspond to real time control requirements. The variable *featFunc* was set in order to extract the following signal features, that were chosen according to previous positive evaluations described in literature: RMS, TD, HIST, mDWT. The *ker* variable was set in order to test parallel computational speed with 2,4,8,16,32 and 48 threads. The server used to run the experiments has 128GB RAM and four Xeon E7-4820 processors, each having 8 cores with Intel Hyper-Threading Technology, which delivers two processing threads per physical core.

When using BioPatRec, the function ExtractSigFeature.m was used to compute both the RMS feature and the concatenation of MAV, MAVS, ZC, SSC, and WL, used to compute the set of time domain statistics described in detail in the paper by Hudgins et al. (1993).

#### Classification

Classification was performed using a Random Forests classifier with 100 trees (Breiman, 2001). The classification is performed on all the movements (rest included) and it is balanced according to the number of movement repetitions. Movement repetitions 1, 3, 4, and 6 were used for training, while repetitions 2 and 5 were used for testing.

## Results

PaWFE^10^ allows to reduce over 15 times the computation time required to extract signal features with the same code (originally based on the Myoelectric control development toolbox and on official MATLAB scripts). In comparison with BioPatRec, computation time reduction is even higher and can easily be over 100 times. PaWFE reproduces the feature extraction part of the sEMG signal classification procedure described by Englehart and Hudgins (2003) in parallel on multiple threads, sensibly reducing computational time. The same classification approach (but in some cases with different signal feature extraction scripts) was applied also in most papers for the characterization of the Ninapro database papers (Atzori et al., 2014a, 2016a,b; Palermo et al., 2017; Pizzolato et al., 2017).

The code was tested on the first 5 Ninapro datasets using 1, 2, 4, 8, 16, 32, and 48 threads. The code allowed to extract RMS, TD, HIST signal features from all the considered datasets (including a total of 50 subjects) in 63 min and all the features in 4.5 h.

The results are summarized in Table 1. Generally, the shortest signal feature extraction times are obtained with 32 cores and the time is equal to 51.86 s per subject and 0.23 ms per time window. These values are 20 times lower than the time required to extract signal features when using a single core in the standard configuration. The fastest signal feature to be extracted is the RMS that takes on average 10.16 s per subject and 0.05 ms per time window (32 threads). The slowest signal feature to be extracted is the mDWT, which takes on average 149.31 s per subject and 0.64 ms per time window. The extraction of the same feature using one thread takes on average 64.92 min per subject and 16.66 ms per time window.

**Table 1:** Feature extraction times for different datasets and features.

			**Parallel time windows pipeline**							**BioPatRec**
		**Features:**	**1 thread**	**2 threads**	**4 threads**	**8 threads**	**16 threads**	**32 threads**	**48 threads**	
Subject feature extraction time (s)	DB1	*RMS*	134.28 ± 2.6	68.79 ± 1.43	36.25 ± 1.06	19.55 ± 0.46	11.07 ± 0.18	7.65 ± 0.27	8.26 ± 0.31	64.69 ± 3.74
		*TD*	189.59 ± 4.53	96.19 ± 2.5	50.18 ± 1.33	26.99 ± 0.53	14.82 ± 0.34	9.71 ± 0.52	11.03 ± 0.74	1971 ± 258
		*HIST*	138.35 ± 3.62	71.18 ± 1.77	37.22 ± 0.89	20.28 ± 0.35	11.19 ± 0.4	7.42 ± 0.64	8.47 ± 0.84	*N.A*.
		*mDWT*	4878.67 ± 156.14	2483.78 ± 53.62	1286.22 ± 25.96	667.7 ± 18.95	343.79 ± 7.16	180.46 ± 6.08	257.56 ± 12.15	*N.A*.
	DB2	*RMS*	102.08 ± 3.17	53.33 ± 0.68	29.63 ± 1.15	17.38 ± 0.27	11.92 ± 0.41	14.04 ± 1.94	17.93 ± 2.56	44.61 ± 1.10
		*TD*	406.96 ± 7.55	208.18 ± 2.31	110.57 ± 3.37	60.47 ± 1.94	34.59 ± 0.53	24.99 ± 1.94	32.65 ± 2.32	540.99 ± 13.79
		*HIST*	189.29 ± 23.62	96.9 ± 8.62	62.26 ± 12.39	39.24 ± 10.36	40.84 ± 12.9	53.53 ± 21.3	49.43 ± 13.87	*N.A*.
		*mDWT*	3696.64 ± 92.36	1880.62 ± 20.75	977.49 ± 15.18	516.65 ± 31.43	265.3 ± 1.95	144.71 ± 3.5	205.29 ± 8.35	*N.A*.
	DB3	*RMS*	97.89 ± 9.82	51.35 ± 4.65	27.96 ± 2.57	16.77 ± 1.61	11.29 ± 1.23	12.64 ± 2.37	16.3 ± 2.67	43.66 ± 2.8
		*TD*	403.68 ± 49.79	195.53 ± 18.9	102.78 ± 10.36	58.06 ± 9.72	32.12 ± 3.25	22.57 ± 3.41	28.46 ± 3.82	734.58 ± 86.71
		*HIST*	185.65 ± 32.79	94.72 ± 14.78	54.27 ± 8.12	36.78 ± 5.44	36.93 ± 12.79	48.51 ± 19	41.63 ± 9.63	*N.A*.
		*mDWT*	3416.13 ± 403.69	1751.21 ± 191.02	910.5 ± 104.92	477.76 ± 67.19	246.34 ± 27.76	134.54 ± 16.29	187.71 ± 24.53	*N.A*.
	DB4	*RMS*	100.14 ± 5.62	53.29 ± 1.61	31.02 ± 6.07	17.62 ± 0.41	12.68 ± 0.54	16.36 ± 2.66	21.3 ± 3.35	43.86 ± 0.70
		*TD*	728.18 ± 11.69	372.47 ± 10.96	194.14 ± 1.01	103.45 ± 1.69	57.42 ± 0.59	38.08 ± 3.13	51.31 ± 3.7	515.92 ± 28.18
		*HIST*	191.55 ± 31.21	100.81 ± 13.21	55.24 ± 9.53	38.22 ± 4.28	42.91 ± 14.75	55.23 ± 22.1	51.65 ± 15.08	*N.A*.
		*mDWT*	3545.72 ± 104.18	1823.32 ± 22.53	949.28 ± 15.55	491.41 ± 9.44	259.15 ± 2.18	142.84 ± 4.09	200.35 ± 9.5	*N.A*.
	DB5	*RMS*	93.57 ± 6.39	48.43 ± 2.4	25.63 ± 1.18	13.95 ± 0.81	7.82 ± 0.65	5.32 ± 0.93	5.76 ± 0.84	45.06 ± 3.35
		*TD*	150.92 ± 10.55	78.45 ± 4.28	41.24 ± 2.52	22.14 ± 1.3	12.31 ± 0.96	8 ± 1.53	9.3 ± 1.36	784.46 ± 117.77
		*HIST*	106.15 ± 9.56	54.88 ± 3.03	28.62 ± 1.59	16.02 ± 0.8	9.15 ± 0.63	7.07 ± 1.24	7.62 ± 1.55	*N.A*.
		*mDWT*	5186.73 ± 392.44	2657 ± 159.61	1386.2 ± 79.69	716.25 ± 36.64	372.74 ± 19.59	193.23 ± 10.37	278.59 ± 12.1	*N.A*.
200 ms time windows (ms)	DB1	*RMS*	0.39 ± 0.005	0.2 ± 0.002	0.105 ± 0.002	0.057 ± 0.001	0.032 ± 0	*0.022 ± 0.001*	0.024 ± 0.001	0.179 ± 0.007
		*TD*	0.55 ± 0.004	0.279 ± 0.003	0.146 ± 0.001	0.078 ± 0.001	0.043 ± 0.001	*0.028 ± 0.002*	0.032 ± 0.002	5.46 ± 0.66
		*HIST*	0.402 ± 0.007	0.207 ± 0.003	0.108 ± 0.001	0.059 ± 0.001	0.032 ± 0.001	*0.022 ± 0.002*	0.025 ± 0.003	*N.A*.
		*mDWT*	14.166 ± 0.296	7.213 ± 0.083	3.735 ± 0.043	1.939 ± 0.039	0.998 ± 0.007	*0.524 ± 0.014*	0.748 ± 0.032	*N.A*.
	DB2	*RMS*	0.491 ± 0.015	0.257 ± 0.003	0.143 ± 0.005	0.084 ± 0.001	0.057 ± 0.002	*0.068 ± 0.009*	0.086 ± 0.012	0.205 ± 0.005
		*TD*	1.959 ± 0.035	1.002 ± 0.011	0.532 ± 0.016	0.291 ± 0.009	0.167 ± 0.003	*0.12 ± 0.009*	0.157 ± 0.011	2.48 ± 0.06
		*HIST*	0.911 ± 0.114	0.466 ± 0.042	0.3 ± 0.06	0.189 ± 0.049	0.197 ± 0.062	*0.258 ± 0.103*	0.238 ± 0.067	*N.A*.
		*mDWT*	17.795 ± 0.448	9.053 ± 0.094	4.705 ± 0.073	2.487 ± 0.154	1.277 ± 0.011	*0.697 ± 0.018*	0.988 ± 0.042	*N.A*.
	DB3	*RMS*	0.49 ± 0.01	0.258 ± 0.006	0.14 ± 0.002	0.084 ± 0.002	0.057 ± 0.003	*0.063 ± 0.009*	0.081 ± 0.009	0.210 ± 0.009
		TD	2.021 ± 0.137	0.981 ± 0.049	0.515 ± 0.024	0.291 ± 0.036	0.161 ± 0.008	*0.113 ± 0.012*	0.142 ± 0.012	3.51 ± 0.21
		*HIST*	0.929 ± 0.132	0.474 ± 0.054	0.273 ± 0.035	0.187 ± 0.038	0.185 ± 0.06	*0.242 ± 0.09*	0.208 ± 0.042	*N.A*.
		*mDWT*	17.127 ± 1.273	8.789 ± 0.637	4.568 ± 0.345	2.394 ± 0.238	1.236 ± 0.088	*0.674 ± 0.049*	0.942 ± 0.093	*N.A*.
	DB4	*RMS*	0.496 ± 0.028	0.264 ± 0.008	0.153 ± 0.03	0.087 ± 0.002	0.063 ± 0.003	*0.081 ± 0.013*	0.105 ± 0.017	0.207 ± 0.003
		*TD*	3.604 ± 0.058	1.843 ± 0.054	0.961 ± 0.005	0.512 ± 0.008	0.284 ± 0.003	*0.188 ± 0.016*	0.254 ± 0.018	2.43 ± 0.13
		*HIST*	0.948 ± 0.154	0.499 ± 0.065	0.273 ± 0.047	0.189 ± 0.021	0.212 ± 0.073	*0.273 ± 0.109*	0.256 ± 0.075	*N.A*.
		*mDWT*	17.547 ± 0.514	9.023 ± 0.112	4.698 ± 0.077	2.432 ± 0.047	1.282 ± 0.011	*0.707 ± 0.02*	0.991 ± 0.047	*N.A*.
	DB5	*RMS*	0.398 ± 0.008	0.206 ± 0.003	0.109 ± 0.003	0.059 ± 0.001	0.033 ± 0.001	*0.023 ± 0.003*	0.024 ± 0.003	0.184 ± 0.006
		*TD*	0.642 ± 0.016	0.334 ± 0.003	0.175 ± 0.003	0.094 ± 0.002	0.052 ± 0.002	*0.034 ± 0.005*	0.039 ± 0.004	3.19 ± 0.39
		*HIST*	0.451 ± 0.026	0.233 ± 0.004	0.122 ± 0.002	0.068 ± 0.001	0.039 ± 0.001	*0.03 ± 0.004*	0.032 ± 0.006	*N.A*.
		mDWT	22.044 ± 0.678	11.301 ± 0.198	5.897 ± 0.14	3.048 ± 0.066	1.586 ± 0.012	*0.822 ± 0.022*	1.187 ± 0.058	*N.A*.

*Subject averages and standard deviation for the entire data acquisitions (in seconds) and for each time windows (in ms). The BioPatRec column reports the results obtained for BioPatRec. The “1 thread” column corresponds to the results obtained without using the parallel window feature extraction algorithm, thus it corresponds to the time required by the original scripts (i.e., the ones included in the Myocontrol Development Toolbox for RMS and TD or the official MATLAB scripts for HIST and mDWT. N.A. stands for “Not available,” meaning that the specific feature is not available within the BioPatRec framework*.

**Figure 2:**
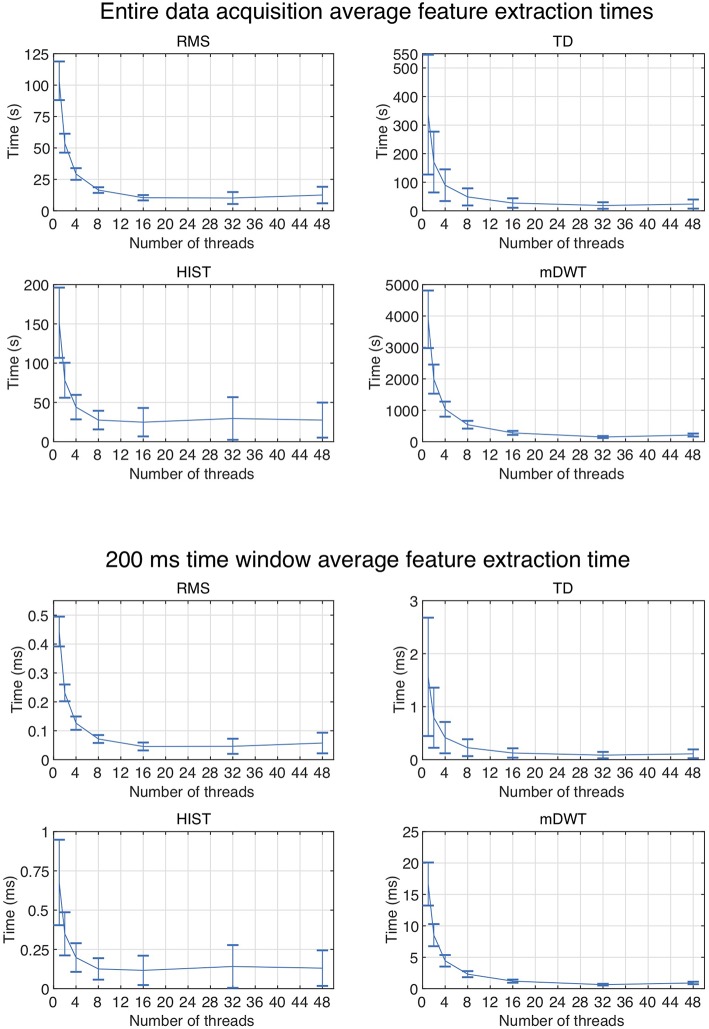
Average subject and time window feature extraction time for all the considered datasets, each signal feature and each test using multiple threads.

**Figure 3:**
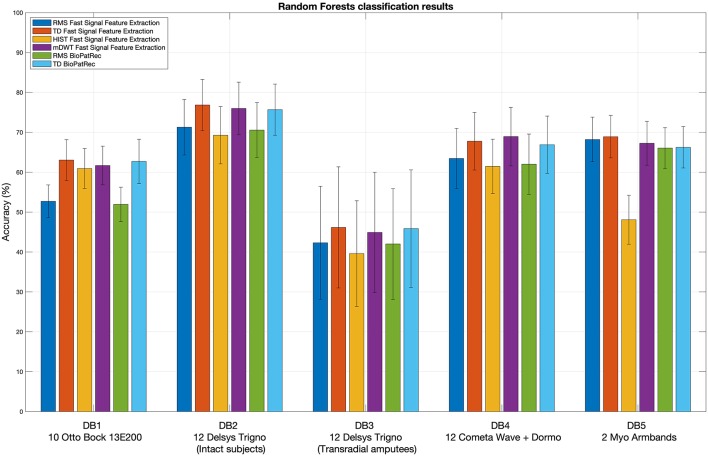
Extracted signal features validation: random Forests classification results obtained using the considered signal features. The histogram includes the classification accuracy obtained for the signal features extracted using BioPatRec as reference.

Figure 2 represents the reduction of each signal feature extraction time considering all the datasets together. Increasing the number of cores reduces the computation time almost linearly, so the scripts allow a considerable reduction of the computation time, also when CPUs with few cores (e.g., 2, 4 or 8) are available. Figure 3 summarizes the average classification accuracy obtained for each dataset and each feature, including also the outcome for features extracted using BioPatRec. The results correspond to results previously described in literature, they are comparable when the features are extracted with the two different algorithms and contribute to validate the quality of the feature extraction scripts.

## Discussion

The code presented in this paper represents a powerful tool for the scientific community. While several studies previously targeted feature extraction in sEMG, to our knowledge there is no software available that can extract signal features in parallel windows from sEMG. Therefore, with previous methods, signal feature extraction from big datasets can easily take several weeks of computational time. The PaWFE innovative approach based on parallel time windows easily reduces of over 15 times the computational time required by signal feature extraction and it allows to extract in few seconds signal features from several hours of sEMG data acquisitions, leading to fast and easy data analysis of big datasets. The approach allows also to extract signal features from 200 ms time windows in <0.1 ms, so it can improve real time data analysis approaches too. PaWFE currently allows to extract 10 different signal features from previous literature and widely used in biomedical signal processing (Chan and Green, 2017), but it can easily be improved with other ones any user. The performance of the code was compared with two widely used feature extraction algorithms: Biopatrec (Ortiz-Catalan et al., 2013), and the Myoelectric Control Development Toolbox^11^ (Chan and Green, 2017), confirming that the proposed approach is effective in reducing the feature extraction time and that the resulting features classification performance are comparable.

With our data analysis setup, RMS and HIST feature extraction time increases in average and variability when using more than 16 threads. This is probably due to the fact that the CPUs need access to more RAM and may saturate it, leading the system to use swap memory (which has lower access speed) for some processes. As well, other possible reasons may be related to hyper-threading of the available 32 cores or to small chip cache.

The outcome of the feature extraction and classification procedure was inserted to provide an example of the code usage and to show that the feature extraction scripts provide results that correspond to other scientific works. However, both the feature extraction procedure and the used classifier influence the classification accuracy. While the use of Random Forest was sufficient to provide an example of the code usage and to show that the feature extraction scripts provide results that correspond to the state of the art, a more thorough benchmarking of different classification methods (including e.g., Support Vector Machines, Linear Discriminant Analysis and Convolutional Neural Networks) is available in previous papers by the authors (Atzori et al., 2014a,b, 2016a,b; Pizzolato et al., 2017).

The use of the code described in this paper can have limitations that are related to the computer hardware and data size. The exact identification of these limits is actually not easy to determine, however at least the following three parameters can be relevant: sEMG data size, number of CPU cores, size of the Random Access Memory (RAM), and RAM clock speed. Parallel data analysis should be performed only with a number of processes that is inferior to the number of CPU cores available. The sum of the memory used by the process for each time window should be inferior to the random access memory available to the computer (128 GB in our case). The tests described in this paper were performed on 16 bit sEMG raw data recorded at 2 KHz, often having size bigger than 400 MB. The feature extraction process worked seamlessly on the workstations described in section Algorithm validation experiments.

The scientific community can directly profit from the code for several reasons. First, the code is publicly available on the Ninapro website, so it can easily and quickly empower new data analysis experiments. Second, thanks to the quick computation, the code allows to perform cross-dataset and cross-procedure comparisons in order to standardize the results across datasets and data analysis procedures. Finally, the code can be extended easily with new and innovative signal features thus also enlarging the code base of the system. Currently, we are extending it to include and parallelize the fused Time-Domain Descriptors signal feature extraction (fTDD, which demonstrated excellent performance for the classification of hand movements in sEMG data, Khushaba et al., 2016), but future contributions from other research groups are also welcome and can be useful to parallelize and accelerate most signal feature extraction procedures.

## Data Availability

Publicly available datasets were analyzed in this study. This data can be found here: http://ninapro.hevs.ch.

## Author Contributions

MA wrote the code, the manuscript, and performed the analysis. HM wrote the manuscript.

### Conflict of Interest Statement

The authors declare that the research was conducted in the absence of any commercial or financial relationships that could be construed as a potential conflict of interest.

## Footnotes

^1^http://www.sce.carleton.ca/faculty/chan/index.php?page=matlab/

^2^The BioSig Project. [http://biosig.sourceforge.net/index.html]

^3^http://www.sce.carleton.ca/faculty/chan/index.php?page=matlab/

^4^http://ninapro.hevs.ch/code/ *(username and password for reviewers: reviewers; rev2019)*.

^5^http://ninapro.hevs.ch/ - last access in June 2018.

^6^http://www.ottobock.com/- last access in July 2018.

^7^https://www.delsys.com/ - last access in July 2018.

^8^http://www.cometasystems.com/ - last access in July 2018.

^9^http://www.thalmic.com/ - last access in July 2018.

^10^http://ninapro.hevs.ch/code/

^11^http://www.sce.carleton.ca/faculty/chan/index.php?page=matlab/
